# Case report: A challenging case of mixed-variant myofibroblastoma with complex imaging and pathological diagnosis

**DOI:** 10.3389/fonc.2024.1438162

**Published:** 2024-10-18

**Authors:** Tomonori Kawasaki, Jiro Ichikawa, Satoshi Kanno, Kojiro Onohara, Masanori Wako, Rikito Tatsuno, Satoshi Ochiai, Takuya Watanabe, Tomoaki Torigoe

**Affiliations:** ^1^ Department of Pathology, Saitama Medical University International Medical Center, Saitama, Japan; ^2^ Department of Orthopaedic Surgery, Interdisciplinary Graduate School of Medicine, University of Yamanashi, Yamanashi, Japan; ^3^ Department of Radiology, Interdisciplinary Graduate School of Medicine, University of Yamanashi, Yamanashi, Japan; ^4^ Department of Orthopaedic Surgery, National Hospital Organization (NHO) Kofu National Hospital, Kofu, Yamanashi, Japan; ^5^ Department of Orthopaedic Oncology & Surgery, Saitama Medical University International Medical Center, Saitama, Japan

**Keywords:** differential diagnosis, MRI, myofibroblastoma, variant, pathological findings, wrist joint

## Abstract

Myofibroblastomas are benign mesenchymal tumors that frequently occur in the groin. They show variable morphology, and the differential histopathological diagnoses are broad, including lipomatous to myxoid tumors. In addition, both pathological and imaging findings may be complex, which makes diagnosis challenging. We herein present a case of a mixed-variant myofibroblastoma of the wrist in a 73-year-old woman. Considering the long clinical course of more than 20 years and the imaging findings, a benign myxoid tumor including a schwannoma was suspected; however, the histopathological findings from resected specimens suggested a diagnosis of myxofibrosarcoma. Additional histopathological findings led to a diagnosis of mixed-variant myofibroblastoma. The differential diagnosis of myofibroblastoma extends beyond imaging to pathological findings because of the number of possible variants. This case reinforces the notion that the gold standard treatment for soft tissue tumors is to perform surgery only after determining the correct diagnosis by biopsy.

## 1 Introduction

Myofibroblastoma (MFB) is a rare benign mesenchymal tumor first identified in the mammary gland in 1987 (1). Initially, the predilection site was thought to be the mammary gland; however, it can occur in various locations throughout the body, including the inguinal region and lower extremities (2). Pathologically, MFBs are composed of bland, short spindle cells with varying degrees of hyalinized collagen bundles; several variants such as lipomatous and myxoid are observed as well (3). Histopathological and molecular genetic findings include CD34 positivity and deletion of 13q14; however, these findings overlap with several tumors, including spindle cell lipoma (SCL), occasionally making the pathological diagnosis difficult (3, 4). The diversity in histopathological findings is accompanied by complex imaging findings, which further complicates diagnosis by imaging. Magnetic resonance imaging (MRI) and computed tomography findings reflect the fatty component present in the tumor, visible as high intensity in the T1 signal, whereas myxoid and collagenized/fibrous tumors show low T1 signal, reflecting the lower fat content (5, 6). Therefore, the differential diagnosis from MRI is broad, and it is extremely difficult to achieve an accurate diagnosis. Herein, we report a case of a rare myxoid MFB of the wrist joint with difficulty in differential diagnosis by imaging and pathological findings.

## 2 Case description

A 73-year-old woman had an asymptomatic mass in her left wrist joint for 20 years, for which no medical examination was sought. Upon experiencing a gradual increase in mass size, she sought medical attention at a nearby hospital, where MRI revealed a benign myxoid tumor suggestive of schwannoma. Marginal resection was performed and postoperative pathological findings suggested myxofibrosarcoma; therefore, she was referred to our hospital for further resection.

**Figure 1:**
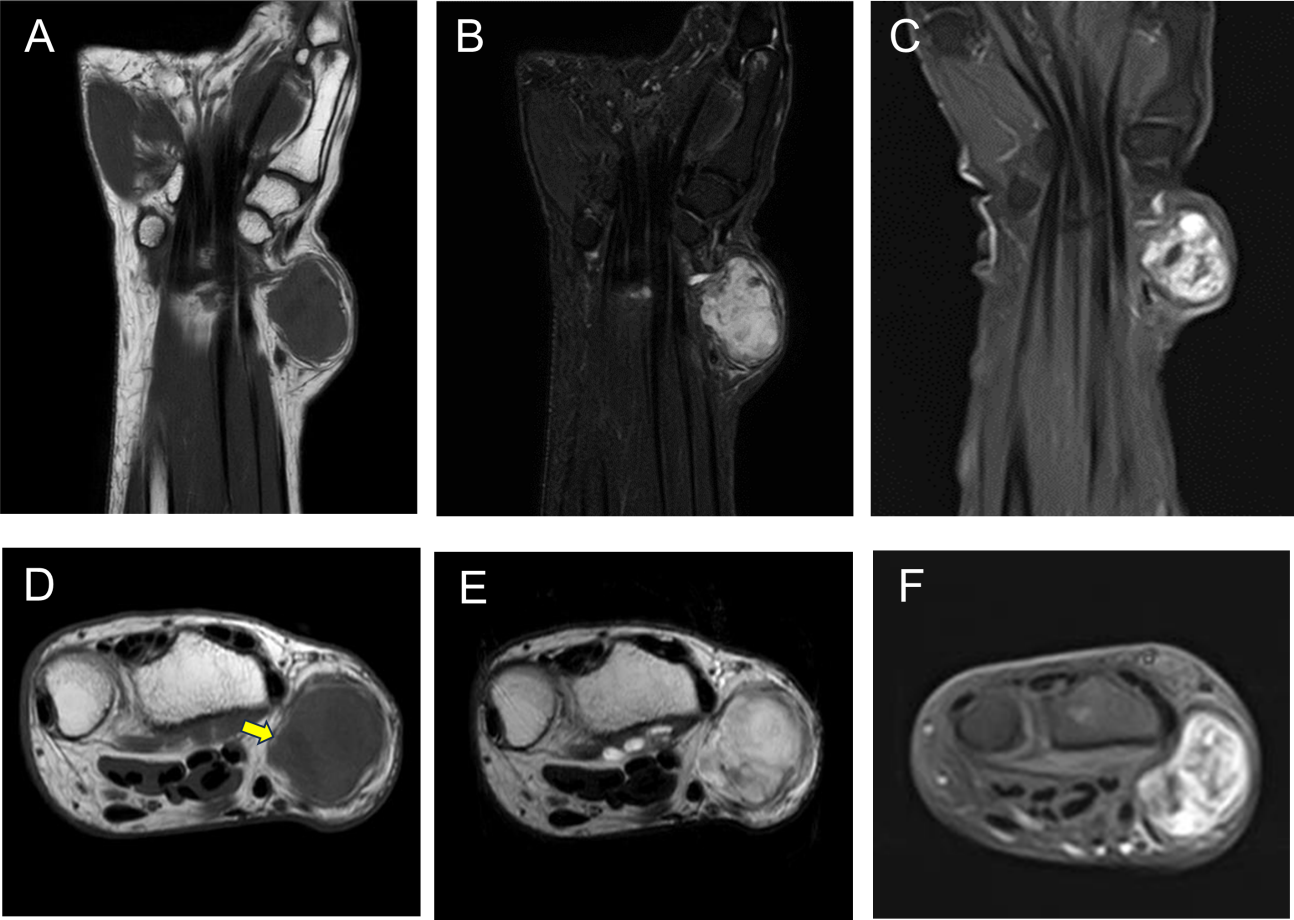
MRI showing an isointense signal on T1-weighted images compared with the muscle **(A, D)**, a high-intensity signal on both STIR **(B)** and T2WI **(E)**, and heterogeneous enhancement on gadolinium-enhanced T1-weighted images **(C, F)**. MRI, magnetic resonance imaging; STIR, short tau inversion recovery; T2WI, T2-weighted imaging.

During the initial visit, no palpable mass was observed, and wrist range of motion was normal. MRI findings from the previous hospital suggested a subcutaneous tumor with isointensity compared to the muscle on T1-weighted imaging (WI) (**Figures 1A, D**) and marked hyperintensity on T2WI and short tau inversion recovery (STIR), with pale hyperintensity resembling walls and septa (**Figures 1B, E**). Gadolinium-enhanced T1WI revealed a heterogeneous enhancement with nodular areas of poor contrast (**Figures 1C, F**). The border between the tumor and surrounding tissues was unclear, with contrast effects that seemed to infiltrate the surrounding fatty tissue (**Figure 1D**, yellow arrow). The MRI findings suggested characteristics of both benign and malignant myxoid tumors. For this case, we only focused on the MRI findings.

## 3 Diagnostic assessment

**Figure 2:**
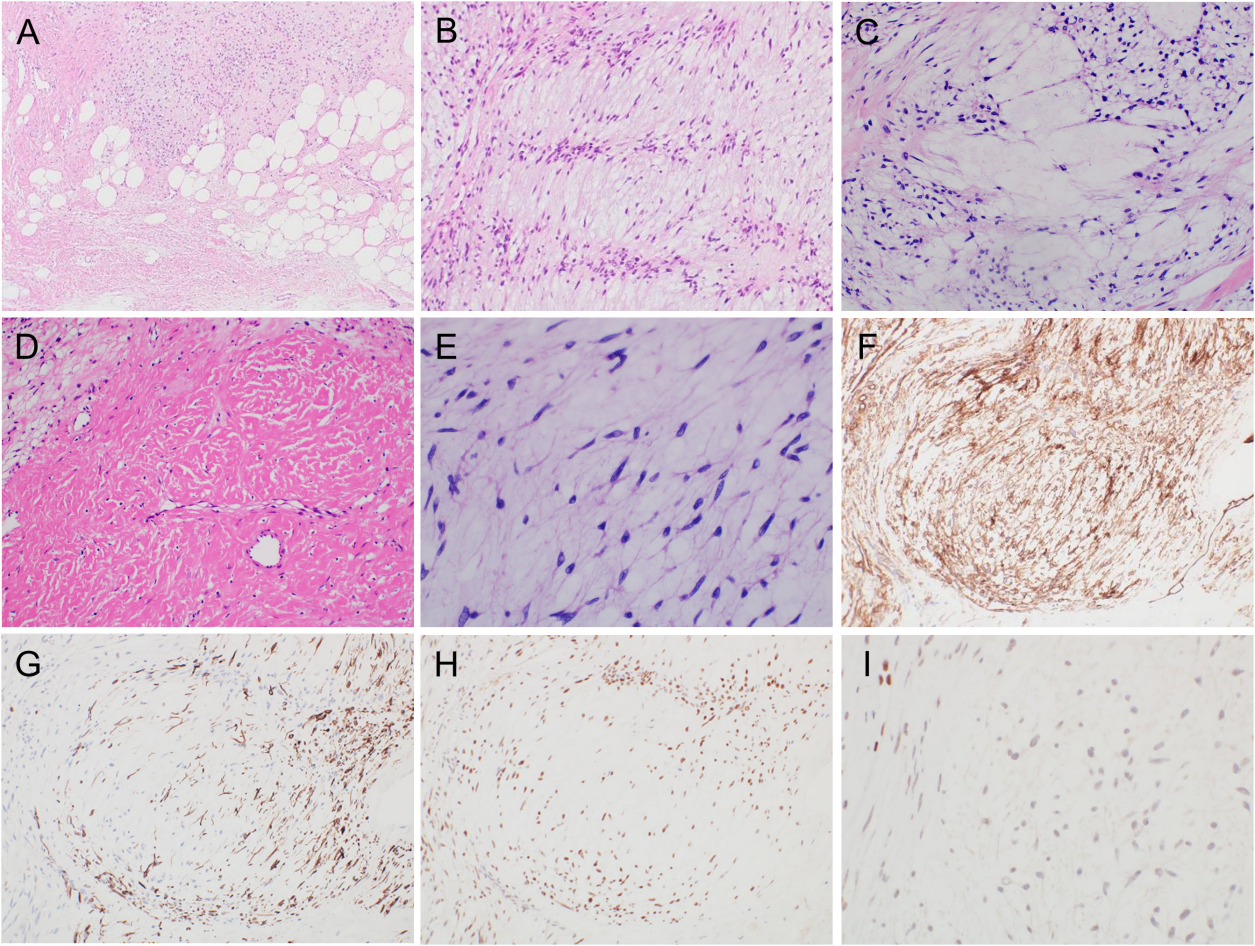
Histopathological findings of the excised specimen. **(A–E)** H&E staining. Immunohistochemical findings for the indicated proteins are shown: CD34 **(F)**, desmin **(G)**, estrogen receptor **(H)**, and Rb1 **(I)**. H&E, hematoxylin and eosin.

Histopathological examination of the excised specimen revealed a flowing growth of spindle-shaped cells with mildly irregular nuclear shapes, increased chromatin in a myxoma-like matrix, and large and small alveolar nest formation (**Figures 2A, B**). Nuclear palisading/Verocay bodies were partially visible (**Figure 2B**). While intranuclear pseudo-inclusion bodies were observed, mitotic activity was not evident. Hyalinization of fibrous tissue was observed in the form of septa. A mixture of lipomatous components was present, with adipocytes displaying slight variations in size, particularly in the peripheral area (**Figure 2A**). Spindled and/or stellate cells set in a loose, edematous, and finely collagenous stroma (**Figure 2C**) as well as sclerotic (keloid-like) collagen fibers were also observed (**Figure 2D**). In a myxedematous background, proliferation of inconspicuously pleomorphic spindle-shaped tumor cells was relatively sparse (**Figure 2E**). Immunohistochemical analysis revealed diffuse CD34 (**Figure 2F**), desmin (**Figure 2G**), and estrogen receptor (ER) (**Figure 2H**) positivity; weak α-smooth muscle actin positivity; and focal loss for Rb1 (**Figure 2I**). While S-100 was partially positive, H3K27me3 expression was maintained. Tests for MDM2, CDK4, and pan-Trk antibodies were negative (data not shown). The Ki67 (MIB-1) labeling rate was less than 5%. Therefore, a final diagnosis of mixed variant MFB was made. As the tumor was diagnosed as benign, only careful follow-up was performed rather than additional surgery.

## 4 Discussion

Soft-tissue MFB was previously considered the counterpart to mammary MFB and was initially thought to occur most frequently in the breast (1, 4). However, the MFB anatomical distribution is wide, and most lesions occur in the groin/inguinal region, followed by the lower extremity, trunk, and breast. The incidence ratio of soft tissue to mammary MFB is 9:1 (2). The age distribution of MFBs is wide, ranging from 4–96 years (mean, 54 years) with a male-to-female ratio of 2 to 1 (2). In addition, the median tumor size is 6.6 cm, and the duration of symptoms ranges from days to more than 20 years. Tumors may even exist for more than 20 years (2). Taking these considerations into account, our case is atypical due to the occurrence in the wrist and in a female patient. In addition to these factors, we highlight the details of pathological and imaging findings which make this case complex.

The essential histological criteria for the diagnosis of MFB include being a purely mesenchymal tumor, presence of hyalinized collagen bundles, low mitotic count, no atypical mitoses, and no necrosis (3). Additionally, several MFB variants, including fibrous, cellular, infiltrating, myxoid, deciduoid, lipomatous, epithelioid, with hemangiopericytoma-like pattern, and atypical, have also been reported (7). Our case showed predominantly myxoid and palisaded/schwannian features with less evident lipomatous features, corresponding to a mixed pattern. In cases of whole and myxoid MFBs, the differential diagnosis based on histopathological findings ranges from benign tumors, such as myxomas and neurofibromas, to malignancies such as myxofibrosarcomas and myxoid liposarcomas (**Table 1**) (8, 9). Both CD34 positivity and deletion of 13q14 represent novel findings in MFBs, and these findings overlap with other tumors (4). Regarding CD34 positivity, this is also seen in SCL, cellular angiofibroma, low-grade malignant peripheral nerve sheath tumors, and dermatofibrosarcoma protuberans, further complicating the differential diagnosis (10). As for 13q14 deletion, including Rb1, distinguishing from SCL, cellular angiofibroma, and atypical spindle/pleomorphic lipomatous tumor (ASPLT) is difficult (11). Based on these findings, SCL is the most difficult to differentiate and shows less desmin positivity than MFB; nevertheless, this positivity is reportedly up to about 25%, suggesting caution is needed for the interpretation of desmin findings (12). Useful IHC findings for identifying MFBs have been reported, including positivity for Vimentin, ER, Progesterone receptor, BCL2, CD10, and CD99, and negativity for cytokeratins, p63, and CD117 (7). In fact, the differential diagnosis between schwannoma and our case is extremely difficult in aspect of morphologic features; however, the positive findings of ER, desmin, and CD34, with heterogenous loss of Rb1, in IHC contributed to the accurate diagnosis.

**Table 1:** Differential diagnoses in myxoid myofibroblastoma.

Benign	Malignant
Myxoma	Myxofibrosarcoma
Nodular fasciitis	Myxoid liposarcoma
Neurofibroma	Mucocele-like tumors (from benign to malignant)
Nodular mucinosis	
Superficial angiomyxoma	

MRI findings are also variable, reflecting the multiple MFB morphological variants (3, 5). In general, a high signal on T1 reflects the presence of fat. For example, there is high signal intensity on T1WI in the MFB lipomatous variant; however, the myxoid and palisaded/schwannian variants show low signal intensity on T1WI (3, 8, 9). In this case, we suspected a myxoid tumor based on the low intensity on T1WI and high intensity on T2WI and STIR. The retrospective analysis of the findings revealed a low fat content at the tumor margins (**Figure 1D**, yellow arrow). It was very difficult to determine whether the fatty tissue was part of the tumor and/or a tumor infiltration. In previous reports of typical cases with a large fat content, i.e., with high intensity on T1WI, distinguishing MFB from liposarcoma, including atypical lipomatous tumors/well-differentiated liposarcoma and dedifferentiated liposarcoma, was important (5). The findings of MDM2 and CDK4 by IHC and fluorescence *in situ* hybridization may provide helpful information to establish correct diagnosis in such cases (11). However, in cases with low fat content, i.e., with a low T1 signal intensity, the differential diagnosis with malignant tumors, including myxoid liposarcoma and myxofibrosarcoma, and benign tumors such as SCL and ASPLT and schwannoma, must be performed. Except for SPL and ASPLT, the differential diagnosis can be made based on the following: i) *FUS* and *EWSR1* rearrangements detected by fluorescence *in situ* hybridization in myxoid liposarcomas (13); ii) no characteristic markers and comprehensive diagnosis in myxofibrosarcoma (14); and iii) the presence of encapsulation and alternating Antoni A and B regions in the schwannoma (9).

In general, the cases with tumors that have been apparent for more than 20 years tend to be diagnosed as benign; however, some sarcomas with an indolent clinical course have also been reported (15). In cases of unplanned excision, which means tumor resection is performed without preoperative diagnosis, additional surgery and plastic surgery reconstruction are often required, placing a heavy burden on the patient (16). In order to avoid such a situation, the process of surgery after an accurate diagnosis by biopsy should be performed. This explains the lack of recurrence after marginal resection and the fact that no cases of metastasis have been reported during the MFB clinical course (8).

In conclusion, we reported a case of mixed-variant MFB in a rare location showing complicated image findings. MFB is worth considering in the differential diagnosis when there is a wide variety of imaging findings and a very broad spectrum of possible diagnoses. Regardless of the size and clinical history, standard treatment is recommended for such lesions after diagnosis by biopsy.

## 5 Patient perspective

Although the long-term clinical course was not indicative of a benign tumor, performing biopsy to establish the correct diagnosis was essential.

## Data Availability

The original contributions presented in the study are included in the article/supplementary material. Further inquiries can be directed to the corresponding author.
